# COVID-19 Related Experiences Among College Students With and Without Disabilities: Psychosocial Impacts, Supports, and Virtual Learning Environments

**DOI:** 10.3389/fpubh.2021.782793

**Published:** 2021-12-10

**Authors:** Darcy Jones McMaughan, Kelley E. Rhoads, Crys Davis, Xuewei Chen, Ho Han, Richard A. Jones, Carlos C. Mahaffey, Bridget M. Miller

**Affiliations:** ^1^Health Education and Promotion, School of Community Health Sciences, Counseling and Counseling Psychology, Oklahoma State University, Stillwater, OK, United States; ^2^Department of Educational Psychology, College of Education and Human Sciences, Oklahoma State University, Stillwater, OK, United States; ^3^Department of Integrative Biology, College of Arts and Sciences, Oklahoma State University, Stillwater, OK, United States; ^4^Arnold School of Public Health, University of South Carolina, Columbia, SC, United States

**Keywords:** COVID-19, students, disability, mental health, higher education

## Abstract

This cross-sectional analysis estimated differences, based on disability status, in college students' (*n* = 777) experiences during the COVID-19 pandemic. Data were modeled using *t*-tests and logistic regression. Most participants were white (86.2%), and women (66.4%). The mode age was 23. A third (35.6%) had at least one disability. Students reported high rates of psychosocial distress, like fear of contracting (59.7%) and spreading (74.3%) COVID-19, worry about friends and family (83.7%), and increased anxiety (72.5%), depression (59.9%), and substance use (24.7%). Forty-two percent (42.2%) were scared they would miss out on their education through virtual classes. About a third feared forgetting assignments (34.1%) and making mistakes (33.9%). Fewer students expressed apprehension about (27.9%) and intimidation by (26.3%) virtual learning. Only 17.2% would continue taking virtual classes after the pandemic. Students with disabilities (*M* = 12.4, *SD* = 4.1) experienced more psychosocial stressors compared to students without disabilities (*M* = 9.9, *SD* = 4.2), [*t*_(775)_ = 7.86, *p* < 0.001]. In adjusted models, disabled students were more than twice as likely to experience worry about medical bills (OR = 2.29), loneliness (OR = 2.09), and increased anxiety (OR = 2.31). They were also more than three times as likely to report increased depression (OR = 3.51) and changes in sexual activity (OR = 3.12). However, students with disabilities (*M* = 1.5, *SD* = 1.1) also reported receiving more support compared to their non-disabled classmates (*M* = 1.1, *SD* = 1.1), [*t*_(775)_ = 6.06, *p* < 0.001]. Disabled students were more likely to feel a sense of contributing to society by following precautions (OR = 1.80) and receive support from family and others (emotional support: OR = 2.01, financial support: OR = 2.04). Interestingly, no significant differences were found in students' feelings associated with online or virtual learning [*t*_(526.08)_ = 0.42, *p* = 0.68]. Students with disabilities, though, trended toward reporting negative experiences with virtual learning. In conclusion, students with disabilities were disproportionately affected by COVID-19 stressors, but also expressed more support and a sense of contributing to the common good.

## Introduction

As community spread of COVID-19 became an increasing concern, college campuses in the United States implemented drastic changes to their day-to-day operations. Students returning to college from spring break were forced to relocate away from campus and engage in alternate learning formats to limit community spread. By the end of March 2020, more than 1,400 colleges and universities had transitioned away from existing course formats relying mostly on face-to-face instruction to virtual learning formats exclusively (1). Almost all supporting institutional infrastructure was modified to decrease in-person contact and slow community spread of the virus. Dormitories and cafeterias shuttered, access to computing laboratories and other educational resources were restricted, and most university staff began working remotely.

Even prior to the challenges brought about by COVID-19, college students in the United States experienced significant levels of psychosocial distress (2). Between 2007 and 2017, the rates of mental health related diagnoses and treatment of mental health conditions among college students in the United States increased by almost 80 and 60%, respectively (3). Changes brought about by the pandemic intensified these psychosocial concerns as students lost social activity, which was one of their main coping strategies (4) and faced upheaval and uncertainty (5, 6). Studies addressing distress among U.S. college students reported, for example, increased or worse depression (7), grief, loneliness, and generalized anxiety (8), stress, and worry (9). While the stressors of the pandemic affected all students, they also intensified long-standing issues for students from historically excluded groups.

Disabled people^1^ are historically excluded from higher education. As Timothy Dolmage asserts in Academic Ableism “disability has always been constructed as the inverse or opposite of higher education” (11). As a symptom of this history of exclusion, people with disabilities account for a quarter (25.7%) of the community population in the United States (12) but only 19% of the undergraduate college student population (13), and are less likely to have completed at least a bachelor's degree compared to people without disabilities (14). Among people aged 25 and older, 40% of those with no disability have at least a bachelor's degree. By that same age, only 20% of their disabled peers completed at least a bachelor's degree (14). Two thirds (67%) of high school students with disabilities graduate on time, a rate lower than the total population (84%) and lower than other historically excluded students. For example, when aggregated by ethnicity, race, and economic status, the graduation rate of Hispanic high school students is 80%, Black high school students is 77%, and economically disadvantaged high school students is 78%—all much higher than disabled students (15). Even when emerging adults with disabilities “make it” to college, their graduation rates drop dramatically at the college level compared to high school, with <35% of disabled college students obtaining a 4-year degree within 8 years of graduating high school (16). There are several factors that directly influence the lower graduation rates among disabled college students. While attending a 4-year college, disabled students report discrimination and barriers to learning. In a large-scale study of 13,844 undergraduates in the United States, Aquino et al. found that 22% of students with disabilities experienced offensive verbal comments (17). Disabled students surveyed in a study of undergraduate students attending Indiana University system colleges also reported lower levels of sense of belonging and higher levels of physical assault, verbal assault, and academic challenges (18). Students with disabilities often cite negative faculty attitudes, like questioning the validity of the student's disability and capability and refusing to adapt classroom techniques, as barriers to success in higher education (19).

Disablism and ableism in the United States further place disabled people in precarious positions with intersecting struggles (20), particularly during emergencies and disasters (21). Disabled people face barriers in accessing appropriate health care (12, 22), discriminatory employment environments (23), impoverishment (24), and stigma (25, 26). COVID-19 precautions lead to disruptions in, for example, much needed community based care and clinical services (27). Health care policies during the pandemic discriminated against disabled people by prioritizing health education, access to medications, services, and life-saving equipment for the abled (28, 29). While disability status is not the same as health status, some disabled people, like those with health conditions associated with poor COVID-19 outcomes or those residing in congregate living settings, were at an increased risk for severe complications and death (30–32).

Even as many factors converged to further hinder the academic progress of disabled college students, little work has been done to explore their experiences in the United States during the pandemic. The current literature focuses primarily on the experiences of personnel and higher education professionals who work with students with disabilities (33, 34), barriers faced by educators who teach students with disabilities (33, 35), and policy analyses (36, 37). The few studies that focused on the experiences of students with disabilities found evidence of hardship and distress during the pandemic. For example, in her narrative reflection, disabled student and advocate, Syreeta Nolan, described the stress of sudden isolation, loss of accommodations and services, and intersectional discrimination due to COVID-19 and long-standing discriminatory practices (38). In a report of undergraduate students at nine universities in the United States, Soria et al., explored well-being among disabled students during COVID-19 looking at, among other factors, financial impact, health during the pandemic, belonging, and engagement (39). According to this report, more disabled students (*n* = 1,788 and representing around 6% of respondents) experienced financial hardships, food insecurity, housing insecurity, lack of safety, and lack a sense of belonging and support on campus than students without disabilities. A greater percentage of students with disabilities also reported major depression and anxiety compared to students without disabilities. However, it is unclear whether these circumstances existed prior to the pandemic or developed because of the pandemic. In another study of COVID-19 experiences, Zhang et al. described the impact of COVID-19 on 28 students with disabilities at the University of Washington, and found that disabled students were more concerned about the switch to virtual learning and experienced more COVID-19 related adversity than students without disabilities (40). Unlike Soria et al., they report that students with and without disabilities experienced similar levels of perceived stress, depression, and anxiety. Similar to Zhang et al., Gin et al. observed that disabled students in STEM (*n* = 66) faced challenges with the move to a virtual learning environment, compound by the loss of accommodations (41).

Following the Spring 2020 semester, many universities utilized the Summer 2020 semester to create campus safety plans for the upcoming Fall 2020 semester to protect the health of students, faculty, and staff and slow the spread of COVID-19. Some universities continued to provide instruction fully online, whereas others provided mostly online instruction with some face-to-face courses having drastically reduced course enrollments to allow for social distancing within classrooms. In addition to adopting state-wide mask mandates, many universities in states without such mandates created their own campus-wide mask mandates while providing both single use and reusable masks to students, faculty, and staff. Some universities reopened a limited number of residence halls and campus dining hubs with special protocols in place for students who needed to quarantine due to diagnosis or exposure to COVID-19. Some institutions even provided support to local, county, and state-level health agencies to aid in contract-tracing and COVID-19 testing efforts.

The current study adds to this very important, but currently limited, literature on the experiences of disabled students in higher education during the COVID-19 pandemic by including measures specifically created to ascertain COVID-19 related-experiences and support and asking students specifically about feelings toward virtual environments. We surveyed students with and without disabilities attending Oklahoma State University in the United States to answer the following research questions:

1. How did students with disabilities experience pandemic-related stress compared to students without disabilities? Based on previous research regarding the experiences of disabled college students during the COVID-19 pandemic (38– 40), we hypothesize that students with disabilities will report significantly higher pandemic-related stressors compared to non-disabled students.
2. Were students with disabilities more likely to receive support during the pandemic compared to students without disabilities? We hypothesize that students with disabilities will report lower feelings of support during the pandemic compared to abled students. This hypothesis stems from the larger body of research regarding the multitude of barriers disabled students face in higher education (16–19).
3. How did students with disabilities perceive online education compared to students without disabilities? Similar to the findings of Gin et al. (41) and Zhang et al. (40), we hypothesize that disabled students in our sample will report more struggles with virtual learning than students without disabilities.

## Materials and Methods

### Study Design

This cross-sectional survey analysis involved estimating differences, based on disability status, in psychosocial impacts, supports, and reactions to virtual learning environments among college students during the COVID-19 pandemic. Full models were adjusted based on social and economic characteristics associated with differences in psychosocial distress. These characteristics included race, ethnicity, gender, income, and rurality (42–44). Report of our study processes and results followed the Checklist for Reporting Results of Internet E-Surveys (CHERRIES) (45).

### Setting

The study took place at Oklahoma State University, a land-grant university with a total enrollment of 24,580 undergraduate and graduate students and located in a rural county in Oklahoma. Oklahoma ranked 12th out of 50 states, Washington DC, and US protectorates for the highest rate of COVID-19 cases per 100,000 people (46). Just prior to the study period, Oklahoma State University made the news when students returning for the fall 2020 semester were filmed partying in packed bars adjacent to the university (47). This study was conducted from August 2020 to December 2020 and was approved by the Oklahoma State University Institutional Review Board (IRB-20-427).

### Positionality

At the time of the study, all the authors taught higher education courses in Oklahoma. Two of the authors are disabled scholars, two of the authors are caretakers for a student with disabilities, and one author grew up with a sibling who was disabled. Their interest in this research and subsequent development of the research questions stems from their experiences in higher education and experiences in the disabled community during the COVID-19 pandemic. COVID-19 disproportionately affected people with disabilities, and they questioned if students with disabilities were also disproportionately affected.

### Participants, Recruitment, and Privacy Protections

All registered students at Oklahoma State University were eligible to participate. Participants were required to be at least 18 years old at the time of the survey. No additional inclusion or exclusion criteria were utilized. Using a targeted and snowball sampling design, a recruitment email linking to the survey was sent to a random sample of 5,000 students to generate a sample of convenience (as is the nature of open surveys). Instructors were also asked to send out the recruitment email to students in their courses. Students who received this email were asked to share the survey link with Oklahoma State University students in their acquaintance circle who had not received the email and were potentially willing to participate. The email included information about the study, the informed consent document, and a link to the electronic survey. The informed consent process consisted of explaining to the student-participants, in plain language, who the principal investigator was, the purpose of the study, the estimated amount of time the survey would take, and the data storage processes. To protect against unauthorized access, all data were collected and stored via a password protected and encrypted professional Qualtrics account. Only investigators with IRB approval had access to the data. The data will be stored for 3 years.

At the end of the survey, student-participants could opt-in to a drawing for a chance to win an Amazon gift card. Gift card prizes were one $100 gift card, two $50 gift cards, and forty $20 gift cards. If participants opted into the drawing, they were redirected to a separate survey and asked to provide an email address for the drawing. This separate survey ensured that survey responses could not be connected to the email provided.

### Data Sources and Measurement

Data were collected as part of a larger study. Of the random sample of 5,000 students who were recruited to participate in the study, 783 students began the survey with 715 of those students completing the survey in its entirety (91% completion rate). An additional 134 students, who were recruited via classrooms and word-of-mouth, completed the study using an anonymous link, which increased our total sample to 849 affirmative responses. However, 72 observations were removed from our sample for missing response for all items, straight line response, speed responses, and for obviously fake or manipulated responses (for example, writing in “Pterodactyl” for gender), resulting in an analytic sample of 777 observations. Most student-participants took between 10 and 25 min to complete the online survey.

The survey was developed by the project research team through an iterative process of item selection and adaptation. Team members first selected existing surveys that measured domains of interest. Scales and items from those surveys were chosen through team discussion and adapted as necessary to fit the population of focus. The final items included a mix of closed items with open options and Likert-type items. The survey domains for this analysis included: (1) demographic data on the student-participant adapted from the National College Health Assessment IIc survey (48) and items created by the research team, (2) COVID related experiences and psychosocial stressors adapted from the Pandemic Stress Index (49), and (3) reactions to virtual learning environments adapted from items in the User Acceptance of Information Technology Scale (50). In the following section we describe the items used in the analysis.

#### Demographic Data

Demographic data items used in this analysis were items adapted from the National College Health Assessment IIc (48) and asked students to report their ethnicity, race, and disability status. Additional items created by the research team asked students to report their gender, zip code of permanent residence, and family income. From the National College Health Assessment, students were first asked “Are you Hispanic, Latinx, or of Spanish origin” and then asked to disclose their racial identification by checking all that applied: White, Black or African American, American Indian or Alaska Native, Asian, and Native Hawaiian or Pacific Islander. Students were also asked to identify their disability status (“Have you ever been diagnosed with any of the following?”) by selecting all applicable options: sensory impairment, mobility impairment, learning disability, mental health disorder, or a disability/impairment not listed. To compare disabled students to students without disabilities, we created a variable indicating the presence or absence of a disability [disability: yes (1)/no [0]] based on an affirmative response to one or more of the conditions listed in the disability item.

Gender, zip code of permanent residence and family income were items created by the research team. The gender item asked students “How would you describe your gender” and included three options: male (including transgender men), female (including transgender women), and prefer to self-describe as (non-binary, gender-fluid, agender, etc.) with an open answer response. Rurality was identified using the self-reported zip code of the student's permanent address and categorized based on Rural-Urban Commuter Area (RUCA) codes (USDA ERS, 2020). RUCA codes classify U.S. census tracts on a continuum of rural to urban categories using patterns of daily commuting, population density, and measures of urbanization. RUCA classifications can be applied from census tracts to ZIP codes. Whether a student originated from a rural community was determined based on permanent residence in a ZIP code associated with large rural, small rural, or isolated RUCA codes.

#### Psychosocial Stressors

Items indicating psychosocial stressors were adapted from the Pandemic Stress Index, which was created at the University of Miami in Florida to measure behavior changes and stress associated with the COVID-19 pandemic (49). More information about the Pandemic Stress Index can be found at the University of Miami School of Nursing and Health Studies Center of Excellence for Health Disparities Research (51). The Pandemic Stress Index included three sets of questions: (1) what behaviors participants have done in the past or are currently doing in “check all that apply” format, (2) how much the COVID-19 pandemic has impacted participants' daily life in Likert-scale format, and (3) what experiences participants have had or are currently having in “check all that apply” format. For our study, we were interested in the last question set and incorporated minor adaptations to include circumstances specific to the college student population (e.g., worry about missing my classes if I get sick with coronavirus), which were not included in the original Pandemic Stress Index. As such, we asked student-participants to report “which of the following are you experiencing (or did you experience) during COVID-19 (coronavirus)?” with a checklist of 24 items measuring experiences of emotional distress, substance use, sexual behavior, financial stress, stigma, support, and “other difficulties or challenges” with an opportunity for participants to provide a descriptive open-ended response. The PSI items were split to measure two different domains for this study: (1) psychosocial stressors and (2) support. Twenty items were grouped together to measure the domain of psychosocial stressors. A few of these items included “more depression,” “fear about the economy,” “stigma or discrimination from other people,” and “increased alcohol or other substance use.” Depending upon whether the participant checked or did not check the box for an item, items were scored dichotomously to represent presence [yes (1)] or absence [no [0]] of the experience. Taken together, a participant's score on psychosocial stressors could range from 0 to 20 based upon the number of boxes they checked indicating the COVID-19 related experiences they had in the past or are currently having.

#### Support During COVID-19

Three items from the Pandemic Stress Index were used to measure the “support” domain. These items asked participants to endorse whether they were “getting emotional or social support from family, friends, partner, counselor, or someone else,” “getting financial support from family, friends, partner, counselor, or someone else,” and “feeling that I was contributing to the greater good by preventing myself and others from getting coronavirus” during the pandemic. The support items were scored similarly to the psychosocial stressors items with participant's scores ranging from 0 to 3.

#### Reaction to Virtual Learning Environments

The remaining outcome measure, reactions to virtual learning environments, consisted of six items adapted from the User Acceptance of Technology Scale to measure reactions to virtual learning (50). These items asked student-participants how much they agree on a scale of 1(strongly disagree) to 7 (strongly agree), with statements related to apprehension, fearing missing out on their education by taking virtual classes, fear of forgetting assignments, afraid of making mistakes they cannot correct, and intimidation around virtual learning environments. The scale includes a reverse-coded item that asked students if they intended to take virtual classes once COVID-19 concerns had passed. Participant scores on this scale could range from 6 to 42. For some of the analyses we conducted (see below), scale items were dichotomized [presence of reaction: yes (1) no [0]]. Responses “agree” and “strongly agree” were re-coded to indicate ‘yes' whereas responses “strongly disagree,” “disagree,” “somewhat disagree,” “neither agree nor disagree,” and “somewhat agree” were re-coded to indicate ‘no' [strongly disagree to somewhat agree = no [0], agree and strongly agree = yes (1)].

### Statistical Analysis

Data were inspected for missing response, straight line responses, speed responses, and fake or manipulated responses. Data were modeled using *t*-tests and logistic regression, which adjusted for the sociodemographic characteristics of race, ethnicity, gender, rurality, and income.

#### T-Tests

Scale scores were used to identify group differences in psychosocial stressors during COVID-19, support during COVID-19, and feelings regarding the virtual learning environment between students with and without disabilities.

#### Logistic Regression Models

We fit logistic regression models for categorical responses (STATA *logit*) to the data for each COVID-19 psychosocial stress, support, and virtual learning environment item. The models included disability status and controlled for socio-economic and demographic characteristics (race, ethnicity, gender, income, and rurality). Logistic regression analyses were conducted using Stata 15 (52). Odds ratios (ORs) and 95% confidence intervals (CIs) are presented.

## Results

### Description of the Student Participants

Table 1 shows the demographic characteristics of the students who participated in the study (*n* = 777). Most of the student-participants were white (86.2%), women (66.4%), and between 18 and 24 years old (79.5%). Just over a third (35.6%) of the students were from rural communities. Among the students who participated in the survey, just over a third (35.6%) reported a disability. About a third (28%) of the students with disabilities reported multiple disabilities.

**Table 1:** Sample characteristics (*N* = 777).

**Demographics**	***n* (%) unless otherwise specified**
Age (Mode, IQR)	21 (19, 23)
Gender (Female)	516 (66.4)
Race/ethnicity^*^
White	670 (86.2)
American Indian or Native American	111 (14.3)
Hispanic/Latino	62 (8.0)
Black	53 (6.8)
Asian	38 (4.9)
Native Hawaiian or Pacific Islander	9 (1.2)
Multiple selected	102 (13.2)
Disability (reported at least one disability)	277 (35.6)
Household income
< $20,000	105 (13.5)
$20,000–$34,999	80 (10.3)
$35,000–$49,999	103 (13.3)
$50,000–$74,999	157 (20.2)
$75,000–$99,999	112 (14.4)
Over $100,000	210 (27.0)
Location of permanent address (rural)	277 (35.6)

*Percentages based on non-missing responses*.

* *Respondents could select more than one option. Percentages will not sum to 100%*.

### Overall Experiences of the Student Participants

Table 2 presents COVID-19 related psychosocial stressors, support, and feelings about virtual learning environments across the entire sample of students who participated in the survey. We found high rates of psychosocial distress among the sample of students. When looking at psychosocial impact, three quarters of the students endorsed concern about transmitting COVID-19 to others (74.3%), and over half (59.7%) feared contracting COVID themselves. The students were worried about friends, families, partners, neighbors, and others (83.7%), missing class should they contract COVID-19 (62.8%) and the economy (58.9%) but were less worried about rates in their community (53.1%), missing work (54.3%) or medical bills (39.5%). A substantial number of the sample of students reported boredom (77.4%), frustration (75%), increased anxiety (72.5%), sleep changes (64.4%), loneliness (61.3%), and increased depression (59.9%) during the COVID-19 outbreak. A quarter (24.7%) of the students said their alcohol or substance use increased because of the pandemic. About twenty percent (18.6%) reported changes in sexual activity.

**Table 2:** All students' experiences during the COVID-19 pandemic (*N* = 777).

**Factors**	***n* (%)**
Psychosocial Stressors
Fear of getting COVID-19	464 (59.7)
Fear of giving COVID-19 to someone else	578 (74.3)
Confusion about what COVID-19 is, how to prevent it, or why social distancing/isolation/quarantines are needed	257 (33.1)
Fear about the economy	458 (58.9)
Worry about friends, family, partners, neighbors, etc.	651 (83.7)
Worry about medical bills if I get sick from COVID-19	307 (39.5)
Worry about missing classes if I get sick from COVID-19	488 (62.8)
Worry about missing work if I get sick from COVID-19	422 (54.3)
Worry about infection rates of COVID-19 in my community	413 (53.1)
Increased depression	466 (59.9)
Increased anxiety	564 (72.5)
Loneliness	475 (61.3)
Frustration	583 (75.0)
Boredom	602 (77.4)
Increased alcohol or other substance use	192 (24.7)
Changes in sexual activity	145 (18.6)
Changes to normal sleep pattern	501 (64.4)
Stigma or discrimination from other people	240 (30.8)
Personal financial loss	380 (48.9)
Not having enough basic supplies	182 (23.4)
Support
Receiving increased emotional or social support	327 (42.0)
Receiving increase financial support	235 (30.2)
Feeling that I was contributing to the greater good by preventing myself or others from getting COVID-19	389 (50.0)
Virtual learning
Feeling apprehensive about virtual learning	217 (27.9)
Feeling intimidated by virtual learning	205 (26.3)
Scared to miss out on education by taking virtual classes	328 (42.2)
Fear of forgetting assignments for virtual classes	265 (34.1)
Afraid of making uncorrectable mistakes in virtual classes	264 (33.9)
Continue to take virtual classes after COVID-19 pandemic	134 (17.2)

*Items measuring psychosocial stressors and support were adapted from Pandemic Stress Index and items measuring virtual learning were adapted from User Acceptance of Information Technology Scale*.

Some students experienced additional adversities during the COVID-19 pandemic. Almost a third (30.8%) of the students who participated in the study reported people treating them differently because of their identity, having symptoms, or other factors related to COVID-19. Almost half (48.9%) reported personal financial loss, and almost a quarter (23.4%) did not have enough basic supplies like food, water, medications, and a place to stay.

Regarding reactions to virtual learning environments, students expressed negative feelings toward taking virtual classes. Across the entire sample of students, forty-two percent (42.2%) said they agreed or strongly agreed that they were scared they would miss out on their education if their classes were virtual. Rates of fear of forgetting assignments and making mistakes were 34.1% and 33.9%, respectively. About a quarter reported “agreed” or “strongly agreed” that they felt apprehensive about virtual learning (27.9%), and that virtual learning is intimidating to them (26.3%). Only 17.2% agreed or strongly agreed that they would continue taking classes virtually once the pandemic ended.

Not all experiences were negative. As far as positive aspects of experiences during the COVID-19 pandemic, half (50%) reported feeling like they were contributing to “the greater good” by following CDC guidelines to prevent the spread of COVID-19. A little less than half reported receiving emotional and social support (42%) and about a third (30.2%) reported receiving financial support from friends, family, and others.

### Comparisons of Experiences of Disabled and Non-disabled Students

Comparisons between students with disabilities and those without disabilities on COVID-19 related psychosocial stressors, supports, and feelings about virtual learning environments are depicted in Tables 3, 4. Overall, disabled students experienced more psychosocial stressors, were significantly more likely to have experienced key stressors, but were also more likely to receive support. There were minimal differences between students with and without disabilities in feelings about virtual learning environments.

**Table 3:** Disability status comparisons using Independent Samples *T*-tests.

	**Students with** **disabilities**		**Students w/o** **disabilities**		
**Factors**	**M**	**SD**	**M**	**SD**	***t*-test**
Psychosocial stressors	12.36	4.13	9.89	4.24	7.86^*^
Support	1.53	1.08	1.05	1.05	6.06^*^
Virtual learning	4.38	1.74	4.33	1.59	0.42

* *p < 0.001*.

**Table 4:** Adjusted likelihood of experiences during COVID-19 pandemic between students with (w/) disabilities (*n* = 277) and students without (w/o) disabilities (*n* = 500).

**Factors**	**Students w/disabilities *n* (%)**	**Students w/o disabilities *n* (%)**	**Adjusted odd ratio (CI)**
Psychosocial stressors			
Fear of getting COVID-19	185 (66.7)	279 (55.8)	1.58 (1.15–2.17)^**^
Fear of giving COVID-19 to someone else	225 (81.2)	353 (70.6)	1.81 (1.25–2.62)^**^
Confusion about what COVID-19 is, how to prevent it, or why social distancing/isolation/quarantines are needed	82 (29.6)	175 (35.0)	0.77 (0.56–1.07)
Fear about the economy	178 (64.2)	280 (56.0)	1.41 (1.04–1.92)^*^
Worry about friends, family, partners, neighbors etc.	253 (91.3)	398 (79.6)	2.80 (1.72–4.54)^**^
Worry about medical bills if I get sick from COVID-19	145 (52.3)	162 (32.4)	2.29 (1.67–3.14)^**^
Worry about missing classes if I get sick from COVID-19	207 (74.7)	281 (56.2)	2.26 (1.62–3.13)^**^
Worry about missing work if I get sick from COVID-19	173 (62.4)	249 (49.8)	1.61 (1.18–2.19)^**^
Worry about infection rates of COVID-19 in my community	173 (62.4)	240 (48.0)	1.77 (1.30–2.40)^**^
Increased depression	216 (77.9)	250 (50.0)	3.51 (2.49–4.94)^**^
Increased anxiety	229 (82.6)	335 (67.0)	2.31 (1.59–3.36)^**^
Loneliness	200 (72.2)	275 (55.0)	2.09 (1.52–2.89)^**^
Frustration	221 (79.7)	362 (72.4)	1.48 (1.03–2.12)^*^
Boredom	221 (79.7)	381 (76.2)	1.25 (0.87–1.80)
Increased alcohol or other substance use	89 (32.1)	103 (20.6)	1.82 (1.30–2.56)^**^
Changes in sexual activity	83 (29.9)	62 (12.4)	3.12 (2.14–4.56)^**^
Changes to normal sleep pattern	204 (73.6)	297 (59.4)	1.84 (1.33–2.54)^**^
Stigma or discrimination from other people	93 (72.5)	147 (29.4)	1.19 (0.86–1.65)
Personal financial loss	161 (58.1)	219 (43.8)	1.73 (1.27–2.35)^**^
Not having enough basic supplies	86 (31.0)	96 (19.2)	1.73 (1.21–2.47)^**^
Support			
Receiving emotional or social support	148 (53.4)	179 (35.8)	2.01 (1.48–2.74)^**^
Receiving financial support	112 (40.4)	123 (24.6)	2.04 (1.48–2.82)^**^
Feeling that I was contributing to the greater good by preventing myself or others from getting COVID-19	165 (59.5)	224 (44.8)	1.80 (1.33–2.44)^**^
Virtual learning			
Feeling apprehensive about virtual learning	86 (31.0)	131 (26.2)	1.26 (0.91–1.75)
Feeling intimidated by virtual learning	79 (28.5)	126 (25.2)	1.16 (0.83–1.63)
Scared to miss out on education by taking virtual classes	119 (42.9)	209 (41.8)	1.06 (0.78–1.43)
Fear of forgetting assignments for virtual classes	105 (37.9)	160 (32.0)	1.29 (0.94–1.76)
Afraid of making uncorrectable mistakes in virtual classes	100 (36.1)	164 (32.8)	1.15 (0.84–1.57)
Continue to take virtual classes after COVID-19 pandemic	58 (20.9)	76 (15.2)	1.41 (0.96-2.08)

*Adjusted for race, ethnicity, gender, income, and rurality. Items measuring psychosocial stressors and support were adapted from Pandemic Stress Index and items measuring virtual learning were adapted from User Acceptance of Information Technology Scale*.

* *p < 0.05;*

** *p < 0.01*.

Table 3 shows that a higher percentage of disabled students endorsed feelings of psychosocial distress compared to students without disabilities. In group comparisons we observed that students with disabilities (*M* = 12.4, *SD* = 4.1) reported experiencing more stressors compared to students without disabilities (*M* = 9.9, *SD* = 4.2), [*t*_(775)_ = 7.86, *p* < 0.001]. These differences are significant.

Table 4 presents the adjusted odds of students with disabilities endorsing psychosocial stressors, supports, and feelings about virtual learning environments. After controlling for sociodemographic characteristics, disabled students were 66% more likely to report fear of getting [OR = 1.58, 95% CI (1.15–2.17)] and 81% more likely to report fear of transmitting COVID-19 [OR = 1.81, 95% CI (1.25–2.62)] compared to students without disabilities. They also had 1.41 times the odds of being fearful about the economy [95% CI (1.04–1.92)]. Disabled students were almost three times more likely to worry about friends and family [OR = 2.80, 95% CI (1.72–4.54)], over twice as likely to worry about cost of medical bills [OR = 2.29, 95% CI (1.67–3.14)] and missing class (OR = 2.26, 95% CI (1.62–3.13)], and over 1.5 times more likely to worry about missing work should they contract COVID-19 [OR = 1.61, 95% CI [1.18–2.19)]. They had significantly higher odds of being worried about infection rates of COVID-19 in their community [OR = 1.77, 95% CI (1.30–2.40)] compared to non-disabled students. Students with disabilities were also 1.48 times more likely to report frustration [95% CI [1.03–2.12)], 2.09 times more likely to report loneliness [95% CI [1.52–2.89)], 2.31 times more likely to report increased anxiety [95% CI [1.63–3.27)], and 3.58 times more likely to report increased depression [95% CI (1.59–3.36)]. They were 73% more likely to say they did not have enough basic supplies like food, water, medications, and a place to stay [OR = 1.73, 95% CI (1.21–2.47)] and to report financial loss [OR = 1.73, 95% CI (1.27–2.35)]. Disabled students were 82% more likely to say their alcohol and substance use increased [OR = 1.82, 95% CI (1.30–2.56)] and over 200% more likely to say their sexual activity changed [OR = 3.12, 95% CI (2.14–4.56)] during the pandemic. Although more disabled students endorsed feelings of stigma [72 vs. 29%), after controlling for other social and economic indicators this difference was not significant [OR = 1.19, 95% CI (0.86–1.65)].

Regarding the reception of support during the pandemic, disabled students (*M* = 1.5, *SD* = 1.1) reported having more supportive experiences compared to non-disabled students (*M* = 1.1, *SD* = 1.1), *t*_(775)_ = 6.06, *p* < 0.001. More specifically, students with disabilities were twice as likely to receive emotional [OR = 2.01, 95% CI (1.48–2.74)] and financial support [OR = 2.04, 95% CI (1.48–2.82)] from friends, family, and others. Additionally, they were 80% more likely to feel like they contributed to the greater good by following COVID-19 protocols [OR = 1.80, 95% CI (1.33–2.44)] than students without disabilities.

In comparing disabled students' (*M* = 4.4, *SD* = 1.7) feelings about online learning to students without disabilities (*M* = 4.3, *SD* = 1.6), no significant differences emerged based upon disability status, *t*_(526.08)_ = 0.42, *p* = 0.68. Using the responses “agree” and “strongly agree” to indicate an affirmative response, we did find that students with disabilities were slightly more likely to express apprehension of [OR = 1.26, 95% CI (0.91–1.75)], fear of forgetting assignments [OR = 1.29, 95% CI (0.94–1.76)], fear of making a mistake that cannot be corrected [OR = 1.15, 95% CI (0.84–1.57)] and feel intimidated by [OR = 1.16, 95% CI (0.83–1.63)] the online learning environments the university implemented during the pandemic. They were essentially equally as likely to fear missing out on their education [OR = 1.06, 95% CI (0.78–1.43)] due to online learning, and slightly more likely to continue taking virtual classes after the pandemic [OR = 1.41, 95% CI (0.96–2.08)]. These results, however, were not significant.

## Discussion

College is a time of transitions, life changes, and increased responsibility for emerging adults. In the United States these stressors, along with adverse events and psychosocial issues that existed prior to enrollment, result in high levels of mental distress among college students. Swift and extreme changes to daily life brought about to mitigate community spread of COVID-19 served to exacerbate mental health concerns among all college students and compound distress among students from historically excluded groups. In our study, we explored COVID-19 related experiences among college students with and without disabilities enrolled at Oklahoma State University during the Fall 2020 semester. Our results suggest that experiences of psychosocial stressors were high among the students surveyed, with disabled students having much higher likelihoods of reporting distress than students without disabilities. We found that, regardless of gender, race, ethnicity, income, or rurality, students with disabilities were over three times more likely to report increased depression and over twice as likely to report loneliness and increased anxiety compared to students without disabilities. This distress extended over into financial hardships and worries, with disabled students about 1.5 to 2 times more likely to report worry about the economy, missing work, and medical bills due to COVID-19 and report experiencing personal financial loss.

However, students with disabilities were also more likely to be supported and have positive feelings about following precautions to prevent the spread of COVID-19. Specifically, students with disabilities had two times greater odds of receiving emotional and financial support from friends, family, partners, counselors, or someone else. They were also 80% more likely to feel like they contributed to the “greater good” by following COVID-19 precautions than non-disabled students.

One factor affecting distress may be the change from in-person classes to a virtual learning environment. Students in our study did express fear, intimation, and apprehension around online learning, with little difference between disabled and abled students. However, with only about a quarter of the students reporting negative reactions to online learning, the switch to virtual formats was likely not the main driver of mental distress in our sample.

### Psychosocial Stressors

Findings across the entire sample suggest all students, regardless of their disability status, experienced increased mental health distress at the time of this study. Much of the research regarding the COVID-19 pandemic and college student mental health have had similar findings regarding this topic (Conrad et al., Charles et al., Hagedorn et al., Hoyt et al., to name a few). More specifically, students are reporting experiencing heightened depression, anxiety, worry, and isolation as a result of “stay-at-home” orders, relocation from campus residence (8), switch to online/virtual learning (40), and financial losses (6, 53). Students who have minoritized or marginalized identities, such as race (38, 54), gender (53), sexual orientation (53), and disability status (38–40), are reporting even higher rates of mental health stressors compared to their classmates. In our study, students who reported having a disability experienced more mental health distress compared to their non-disabled classmates. Our findings specifically about depression and anxiety align with Soria et al.'s findings (39). Though our study differed from how Soria et al. grouped students with disabilities (grouped as a whole vs. grouped according to type of disability), both samples of disabled students reported having, at least, more than double the likelihood of experiencing COVID-19 related depression and anxiety compared to students without disabilities. This aligns with reports of mental distress among disabled adults outside of the academic setting. In April and May 2020, Okoro et al. found that adults with any disability type were three times more likely to report depressive symptoms compared to non-disabled participants (55).

Other psychosocial stressors that students with disabilities experienced at significantly higher rates compared to non-disabled students were related to financial hardships. More specifically, disabled students in our sample were 40% more likely to report being afraid for the economy, 60% more likely to be worried about missing work if they contracted COVID-19, almost twice as likely to experience personal financial losses, and over two times more likely to worry about having to pay medical bills. Other researchers reported similar findings as it relates to financial distress and hardships among disabled college students. For example, disabled participants in Soria et al.' study were twice as likely to report losing wages from their off-campus employment (39). Additionally, Zhang et al. found personal financial loss to be significantly higher among students with disabilities (40). Interestingly, among the general population of adults, Okoro et al. found non-disabled persons were significantly more likely to report experiencing job or income loss compared to adults with disabilities (55). These findings may contrast with our study due to the differences in how these data were collected and reported. Okoro et al.'s window of data collection was smaller (April–May 2020) compared to the current study because students could report on past and current experiences of financial hardships going back to March 2020 through the administration of the survey (August–December 2020).

In addition to experiencing more financial hardships, students with disabilities reported not having adequate access to the resources they needed during the pandemic. In our sample, disabled students were almost 75% more likely to not have basic supplies such as food, water, medications, and a safe place to live. Though we did not have separate items measuring lack of or worry about food and housing, our findings are still similar to previous research that measured these topics specifically. Soria et al. found similar results, with disabled students in their study being three times more likely to experience food insecurity compared to non-disabled students, and depending on disability type between 1.5 and 3.5 times more likely to experience unexpected spending for technology (39). Similarly, adults with disabilities were 50% more likely to worry about not getting enough food as compared to adults without disabilities (55). Regarding housing security, disabled students were twice as likely (39) and disabled adults were 70% more likely (55) to experience housing insecurity or instability as compared to non-disabled individuals. In March 2020, many universities closed residence halls and required students to find housing elsewhere with relatives or friends. Conrad et al. reported students experiencing more grief, loneliness, and anxiety over having been mandated to relocate (8). Taken together, an intermingling of psychosocial stressors, such as health concerns regarding COVID-19, employment instability, financial hardships, and food and housing insecurity, created experiences of psychosocial distress among both disabled and non-disabled students.

### Support

An unexpected, though positive, finding from our study indicated that students with disabilities were more likely to be recipients of support from family, friends, and others compared to students without disabilities. More specifically, we found that students with disabilities were twice as likely to receive financial, emotional, and social support compared to non-disabled students. These findings are particularly interesting as they appear contrary to previous research on such topics. In their survey of students in California, Soria et al. reported that students with disabilities were more likely to have family members who experienced a reduction or loss of income during the pandemic compared to students without disabilities (39). Additionally, disabled students in Zhang et al.'s study also reported higher family financial loss (40). Such financial losses may exacerbate stress levels within households and increase rates of violence and abuse taking place in homes. For example, disabled students from Soria et al.s' study were significantly more likely to be in living situations where they experienced physical or emotional abuse compared to students without disabilities (39). Furthermore, students with disabilities were two to three times more likely to indicate it was “never true” or “sometimes true” that they resided in an abuse-free living situation. Disabled adults, outside of a collegiate environment, have also reported being two and half times more likely to experience physical or emotional abuse (55).

Disabled students in our study were more likely to report psychosocial distress, financial loss, *and* support from people in their community—results that may appear at odds. However, our findings suggest that such experiences are not mutually exclusive. In other words, students with disabilities who are struggling with psychosocial stressors were able to rely upon their social support systems to help alleviate some of the challenges they were facing during the pandemic. Interestingly, even given the increased psychosocial distress experienced by disabled students in our study, they still reported feeling as if they were contributing to the collective “greater good” by following CDC guidelines (e.g., limiting contact with others, not participating in large gatherings or events), more so compared to non-disabled students. This mindset aligns with the body of research that found greater prosocial behavior occurs among people experiencing stressful, marginalizing, and resource-poor environments [see the work of Paul K Piff, starting with Piff et al. (56)].

### Virtual Learning

Interestingly, there were no statistically significant differences in feelings regarding online learning between students with and without disabilities. Most students in our study reported having neutral feelings (selected “neither agree nor disagree”) regarding online classes when asked about their apprehension, intimidation, making mistakes, forgetting assignments, and missing out on educational opportunities. This finding does not align with previous research regarding this topic from previous researchers. When Zhang et al. described the impact of COVID-19 on students with disabilities at the University of Washington, they found that disabled students were more concerned about the switch to virtual learning and experienced more COVID-19 related adversity in learning than students without disabilities (40). Within the interviews conducted by Gin et al., disabled students also voiced concerns regarding online learning as many students were unable to access previously established accommodations or instructors were autonomous in the decision-making of what accommodations were deemed appropriate (41). As such, it is not surprising that students with disabilities reported distress regarding the transition to online learning, course grades, and impact on matriculation and graduation (40, 41). Both disabled and non-disabled students in our sample may not have had heightened concerns regarding online learning because data collection occurred during the Fall 2020 semester. In other words, instructors at this institution had previously dealt with the swift transition to online learning in mid-March 2020 and received institutional support regarding technology and pedagogy over Summer 2020 to increase the quality of online instruction for the Fall 2020 semester.

### Limitations

Findings from our study are not without limitations. Timing is an important variable to consider due to the cross-sectional nature of the survey. Participants completed the survey in the Fall 2020 semester at a time when state-level and campus-wide restrictions were becoming less restrictive. Some counties in the state no longer required masks, and, though campus and local city policies required mask wearing, some academic courses returned to face-to-face instruction, students returned to living in residence halls, and campus activities, such as sporting events, began to take place again. The recommencement of typical college activities and experiences could have led to students reporting less intense distress due to their increase in interactions with friends, classmates, and faculty/staff compared to the Spring 2020 semester when instruction was abruptly shifted online, and campus housing emptied. Furthermore, the wording of the question adapted from the PSI used to assess psychosocial impacts of COVID-19 allowed participants to report both past and current COVID-19 experiences without timing differentiation. In other words, it is unknown if a participant was currently experiencing more depression at the time of survey completion or if they had previously experienced more depression a few months prior to survey completion. Additionally, the demographics of students who participated in the survey were largely homogeneous, and, as such, our findings may not accurately reflect the feelings and experiences of all college students. Furthermore, our rate of disabled students is higher than other national estimates (35 vs. 19%), presumably due to students with disabilities selecting into the study at a higher rate than other students. We also focused on an aggregate measure of disability, rather than differences between different types of disability. While an aggregate measure provided a more robust sample, we must acknowledge the diversity in lived experiences between people with different disabilities, particularly during the COVID-19 pandemic. Within the disability community there are opposing and conflicting experiences of the same pandemic-related factors, and these differences often fall along the lines of type of disability.

### Implications

The most significant findings of our study were associated with factors outside of academic performance, which suggests that disabled students, particularly, experienced compounded stressors during the COVID-19 pandemic that affected their overall well-being. Campuses should take a holistic approach to supporting students with disabilities that strengthens accommodations but is not limited to reducing academic stressors. A holistic approach must be proactively implemented and not simply a reaction to extreme circumstances like natural disasters or a pandemic. Copeland et al. point out that institutional mitigation strategies that promote emotional and behavioral wellness and foster a sense of community were modestly but persistently beneficial for first-year college students during the pandemic, who are perhaps the most vulnerable to the stressors associated with academic life and the rapid changes due to COVID-19 (57). Reaching further, in discussing priorities for mitigating the pandemic's effect on college students' mental health, Liu et al. propose using the COVID-19 crisis as a leverage point to implement innovative models of support that highlight assets and strengths associated with students' identities and factors that promote resilience (58). These strategies could and should be considered regardless of whether college students are facing a global crisis.

However, neither article—one of which speaks directly to higher education administrators—mentions students with disabilities, which serves to highlight the lack of visibility of disabled students in higher education which, in turn, compounds stressors and creates obstacles. For example, while Liu et al.'s suggestions are forward thinking and could serve to reduce barriers to mental health services among college students, disability is missing in this discussion of at-risk students. They recommend helping students “name and claim pre-covid identity factors to promote resilience” but do not mention disability as an identity factor (12, 58). This aligns with Meleo-Erwin et al.'s findings that accessibility information was mostly missing from student services websites during the pandemic, Gin et al.'s findings regarding inaccessibility of established accommodations, and Soria et al.'s findings regarding disabled students' feeling significantly unsupported by their university (36, 39, 41). Together, these findings suggest that disabled students were less than an afterthought during the pandemic.

When considering our findings through a student-identity lens, our results reiterate the importance of disability as an identity factor because after holding other identity factors like race, gender, ethnicity, rurality, and economic status constant, disabled students were still more likely to report psychosocial distress and negative experiences associated with the pandemic. And, perhaps, due to the inadequacy of institutional support for students with disabilities, they were also more likely to seek out and receive emotional and financial support from persons outside of their university. Thus, any changes to current systems to mitigate mental distress among college students, whether during a crisis like the COVID-19 pandemic or otherwise, must take into consideration both the academic and non-academic needs of disabled students and disabled identity as crucial components of support and services in highereducation.

## Data Availability Statement

De-identified data may be made available upon reasonable request.

## Ethics Statement

This study involving human participants was reviewed and approved by the Oklahoma State University Institutional Review Board. The participants provided their written informed consent to participate in this study.

## Author Contributions

DM contributed to conception of the study. CD managed human subjects' protocols. KR organized the database. DM and KR performed the statistical analysis and wrote the first draft of the manuscript. XC and HH reviewed the analytic code and output. All authors contributed to manuscript revision, read, approved the submitted version, and contributed to the design of the study.

## Funding

This project was support by funding from the Joan Donelson Jacques Endowed Professorship in Health Promotion.

## Conflict of Interest

The authors declare that the research was conducted in the absence of any commercial or financial relationships that could be construed as a potential conflict of interest.

## Publisher's Note

All claims expressed in this article are solely those of the authors and do not necessarily represent those of their affiliated organizations, or those of the publisher, the editors and the reviewers. Any product that may be evaluated in this article, or claim that may be made by its manufacturer, is not guaranteed or endorsed by the publisher.

## Footnotes

^1^We alternate between identity-first language and person-first language to fit with the language preferences of the disability community (10).
